# Supplementary material for: How to Create and Evaluate a Resident-Led Audio Program: Six Clinical Podcasts for Medicine House Staff
Source: MedEdPORTAL. 2020 Dec 30;16:11062. doi: 10.15766/mep_2374-8265.11062 (PMC7780742; doi:10.15766/mep_2374-8265.11062)
Supplement: Supplementary file 1 — Needs Assessment Questionnaire.docxPre- and Postsurveys.docxDevelopers Guide.docxCompleted Developers Guide.docxGI Bleed.mp3SVT.mp3Toxidromes Part 1.mp3Transfusion Reactions.mp3Hypoxemic Respiratory Failure.mp3WCT.mp3 [file mep_2374-8265.11062-s001.zip › A. Needs Assessment Questionnaire.docx]

**Medical Ed and Podcasts for Residents**

Please complete the survey below.

Thank you!

What year in training are you?

R1

R2

R3

Other

Please Describe:

__________________________________

From the list below, what clinical scenarios during your internship made you uncomfortable or were particularly

challenging? Click all that apply.

Wide Complex Tachycardias

Undifferentiated Volume Overload

NSTEMI

Transfusion Reactions

Acute Renal Failure

Syncope

Hypoxemic Respiratory Failure

Hypercarbic Respiratory Failure

Diffuse Rash

Delirium

Acute Renal Failure

Acute Liver Failure

Toxidromes and Overdoses

Sickle Cell Crisis

Cystic Fibrosis and Bronchiectasis

Code Stroke

Dirty Urinanalyses

Consent to Treatment

Challenging Family Dynamics

How many hours a week do you listen to podcasts?

I don't routinely listen to podcasts

0-2 hours

2-4 hours

> 4 hours

What is the ideal length of a podcast in your life?

Less than 10 minutes

10-20 minutes

More than 20 minutes

None - there is no ideal length for me

Do you listen to any medical podcasts?

Yes

No

What do/would you appreciate most in a medical podcast? Click all that apply.

Clinical pearls

References to key literature

Expert opinion

Latest or emerging innovations

Other

I see no role for medical podcasts in my life

What else would you appreciate from a medical podcast?

__________________________________

Would you utilize a podcast series designed for internal medicine residents focused on the essentials of managing

topics like those mentioned above?

Yes

No

THANKS FOR TAKING OUR SURVEY
