# Supplementary material for: How to Create and Evaluate a Resident-Led Audio Program: Six Clinical Podcasts for Medicine House Staff
Source: MedEdPORTAL. 2020 Dec 30;16:11062. doi: 10.15766/mep_2374-8265.11062 (PMC7780742; doi:10.15766/mep_2374-8265.11062)
Supplement: Supplementary file 1 — Needs Assessment Questionnaire.docxPre- and Postsurveys.docxDevelopers Guide.docxCompleted Developers Guide.docxGI Bleed.mp3SVT.mp3Toxidromes Part 1.mp3Transfusion Reactions.mp3Hypoxemic Respiratory Failure.mp3WCT.mp3 [file mep_2374-8265.11062-s001.zip › B. Pre- and Postsurveys.docx]

**Example Episode Pre-Survey**

Please complete the survey below. Thank you!

What year in training are you?

R1

R2

R3

Other __________________________________

Please answer the following questions regarding the episode’s topic.

I feel I have a good understanding of the topic discussed above

Strongly Agree

Somewhat Agree

Neutral 
Somewhat Disagree

Strongly Disagree

I feel confident in my ability to manage the above clinical scenario independently

Strongly Agree

Somewhat Agree

Neutral 
Somewhat Disagree

Strongly Disagree

I would feel comfortable providing education (bedside teaching, "chalk talks" etc.) on the above clinical scenario

Strongly Agree

Somewhat Agree

Neutral 
Somewhat Disagree

Strongly Disagree

**Example Post-Survey**

As a participant in this study, please answer the questions below. Thank you!

What year in training are you?

R1

R2

R3

Other __________________________________

Please answer the following questions regarding the episode’s topic

I feel I have a good understanding of the topic above

Strongly Agree

Somewhat Agree

Neutral 
Somewhat Disagree

Strongly Disagree

I feel confident in my ability to manage the above clinical scenario independently

Strongly Agree

Somewhat Agree

Neutral 
Somewhat Disagree

Strongly Disagree

I would feel comfortable providing education (bedside teaching, "chalk talks" etc.) on the above clinical scenario

Strongly Agree

Somewhat Agree

Neutral 
Somewhat Disagree

Strongly Disagree

Would you recommend this podcast to a fellow resident?

Yes

 No

What aspects of the podcast do you find particularly well-crafted or helpful? Click all that apply.

The overall length of the podcast 
The relevance of the clinical pearls 
The amount of supportive evidence provided The level of ease accessing the content 
The dynamic between the hosts 
Other

________________________________

What are some areas of improvement you have identified? Click all that apply.

The podcast was overall too long 
The podcast was overall too short 
The clinical pearls discussed were not clinically helpful 
There was not enough supporting evidence provided

The podcast was too difficult to access/listen to 
The recording quality of the podcast was too low

The format of the content was uninteresting or dry

Other

__________________________________
