# Supplementary material for: How to Create and Evaluate a Resident-Led Audio Program: Six Clinical Podcasts for Medicine House Staff
Source: MedEdPORTAL. 2020 Dec 30;16:11062. doi: 10.15766/mep_2374-8265.11062 (PMC7780742; doi:10.15766/mep_2374-8265.11062)
Supplement: Supplementary file 1 — Needs Assessment Questionnaire.docxPre- and Postsurveys.docxDevelopers Guide.docxCompleted Developers Guide.docxGI Bleed.mp3SVT.mp3Toxidromes Part 1.mp3Transfusion Reactions.mp3Hypoxemic Respiratory Failure.mp3WCT.mp3 [file mep_2374-8265.11062-s001.zip › C. Developers Guide.docx]

**Developer’s Guide to Creating Podcasts**

This guide is organized into 5 steps, or stages of podcast production. Each stage begins with essential **questions**, which will guide working through the **tasks** necessary to complete the **product** of each stage.

**1) Picking a topic:**

**Questions to ask:**

- What are your motivations behind this project?
  - Are you guided by personal interests?
  - Is it aimed at a specific audience of learners?
  - Would you like to design it to fill curricular gaps?
- What needs assessments can inform the plan?
  - What educational resources already exist?
  - Could a survey of the target audience preferences inform content or format choices?

**Tasks to complete:**

- Identify a podcast topic by defining it with a specific clinical scenario.
- Articulate clinical questions prompted by the scenario to ensure that the topic is focused on clinical reasoning and not rote memorization.

**Tangible product:**

- Educational topic for this episode.

**2) Collecting and organizing content:**

**Questions to ask:**

- How do you define your audience of learners?
- What is the scope of your content?
  - What is outside your scope (i.e. what will you not be discussing)?
  - Are you focusing on the initial steps in a clinical scenario? How about follow-up?
  - Is the focus on diagnostic, therapeutic, or prognostic steps?

**Tasks to complete:**

- Reference trusted sources to ensure accurate and timely content.
- Identify clearly defined learning objectives and key points.
- Emphasize key action steps in a narrative timeline of the scenario.
- Avoid content outside the defined scope.

**Tangible Product:**

- Summary of educational content.

**3) Drafting a script**

**Questions to ask:**

- What voices or perspectives are best suited to the educational goals?
  - Would a voice of authority (e.g. attending, specialist) or of a peer be more effective?
  - Would a monologue from an individual be appropriate? Are there advantages in entertainment or educational value to including multiple voices?
- How do the learning objectives create a framework for organizing the educational content?

**Tasks to complete:**

- Utilize one or more narrative arcs to organize the educational content by following the course of a patient, a clinician, or a learner.
- Find or create opportunities for spaced repetition of key concepts.
- Design an introduction and conclusion for engaging the audience, highlighting learning objectives, and thanking contributors.
- Identify a senior advisor with expertise in the topic.
- Send script drafts to the advisor for revision and review.
- Incorporate feedback into the script draft, repeat process for further review as needed.
- Continue the iterative process until a satisfactory script has been created.

**Tangible product:**

- Final script to be used for recording.

**4) Recording audio**

**Questions to ask:**

- What tone do you want to set?
  - Do you want to focus on efficient delivery or have more humorous conversation?
  - Do you want to portray yourself as the authority on a topic, or express uncertainty?
- What is your budget for audio equipment and processing?

**Tasks to complete:**

- Practice aloud and revise the script to suit spoken language and refine the delivery.
- Identify areas poorly suited for the audio format such as lists or dense content. Consider utilizing a framework.
- Send audio recording to expert clinical for final approval.
- Use audio post-production tools to remove unwanted sounds, level audio and increase overall podcast polish.

**Tangible Product:**

- Finalized recording of episode ready for dissemination.

**5) Releasing a podcast**

**Questions to ask:**

- What will be used to measure success?
  - Is the number of downloads a meaningful outcome for the goals of this project?
  - Are pre and/or post attitude or knowledge assessments feasible? Will they be informative?
- What is the material use-case for listeners of this podcast?
  - Will users need to access it at any time or place with minimal barriers? Or should access be restricted for security or tracking access?
  - Are they using it within a structure of planned activities before or after listening?

**Tasks to complete:**

- Choose a podcast hosting site to suit needs and upload audio files. Consider whether the platform will allow it to be searchable on iTunes, hosted on institutional servers, or password protected.
- Distribute announcements about this new content: in person, e-mail, blog post, or social media.

**Tangible product:**

- Finished audio file is available for download or streaming in web browser or application.
