# Supplementary material for: How to Create and Evaluate a Resident-Led Audio Program: Six Clinical Podcasts for Medicine House Staff
Source: MedEdPORTAL. 2020 Dec 30;16:11062. doi: 10.15766/mep_2374-8265.11062 (PMC7780742; doi:10.15766/mep_2374-8265.11062)
Supplement: Supplementary file 1 — Needs Assessment Questionnaire.docxPre- and Postsurveys.docxDevelopers Guide.docxCompleted Developers Guide.docxGI Bleed.mp3SVT.mp3Toxidromes Part 1.mp3Transfusion Reactions.mp3Hypoxemic Respiratory Failure.mp3WCT.mp3 [file mep_2374-8265.11062-s001.zip › D. Completed Developers Guide.docx]

**Example of Developer’s Guide for Creating Podcasts Worksheet for**

**Episode 1 Acute Gastrointestinal Bleeding**

**1) Picking a topic:**

**Questions to ask:**

- What are your motivations behind this project?

*Our personal interests are in hospital medicine and critical care. We intend to educate junior clinicians (interns) with a focus on appropriate triage and management in urgent situations.*

- What needs assessments can inform the plan?

*The intern teaching conference curriculum for core clinical content at our residency program does not cover acute gastrointestinal bleeding. Core Teaching Conference curriculum for residents contains one lecture on acute gastrointestinal bleeding, and repeats every 18 months, but is not mandatory or recorded for future reference.*

*Residents listening to podcasts for medical education from free public sources is anecdotally common but use and format preferences were not known. We conducted a needs assessment survey about topics and format that our target audience would prefer.*

**Tasks to complete:**

- Identify a podcast topic by defining it with a specific clinical scenario.

*Acute GI Bleeding: Rapid response for a significant volume of blood loss by hematemesis, melena, or hematochezia.*

- Articulate clinical questions prompted by the scenario to ensure that the topic is focused on clinical reasoning and not rote memorization.

*What should a resident be focused on during the initial assessment of a patient with an acute GIB? What steps must be taken first? And what should they do in their initial assessment to guide more definitive evaluation or treatment? And why are those the most important steps?*

**Tangible product:**

- Educational topic for this episode.

*Assessment and initial management of acute GIB in the inpatient setting (ED or acute care floor)*

**2) Collecting and organizing content:**

**Questions to ask:**

- How do you define your audience?

*Our audience are our co-residents, particularly PGY-1s.*

- What is the scope of your content?

*We will focus on the initial assessment, diagnostics and therapeutics of discrete clinical scenarios in acute care medicine. Longitudinal management, sub-specialty service decision making and management decisions after the initial clinical scenario are outside of our scope.*

**Tasks to complete:**

- Reference trusted sources to ensure accurate and timely content

*We will reference key pieces of primary literature, as well as online clinical resources (UptoDate, Dynamed). We are also utilizing expert opinion of physicians at the University of Washington.*

- Identify clearly defined learning objectives and key points

*Recognize the key criteria in determining the severity of an acute gastrointestinal bleed*

*List the laboratory tests that should be ordered to assess coagulopathy in a patient with gastrointestinal bleeding and identify the medication needed to correct it.*

*Choose the correct medications to be used in patients with gastrointestinal bleeding, including additional medications needed in the setting of liver dysfunction.*

- Emphasize key action steps in narrative timeline of scenario

*Determine if patient is stable or unstable*

*Obtain adequate access and begin appropriate resuscitation*

*Identify and correct underlying coagulopathy*

*Discern if the patient has liver disease and begin appropriate medical management*

- Avoid content outside the defined scope

*We will not discuss massive gastrointestinal bleeding, endoscopic management options or options for treatment if patient has recurrence of bleeding.*

**Tangible Product:**

- Summary of educational content

*The above steps culminated 7-page summary outline on the key initial management steps of acute gastrointestinal bleeding.*

**3) Drafting a script**

**Questions to ask:**

- What voices or perspectives are best suited to the educational goals?

*We want to provide a voice from senior residents as peers who are identifying the teaching points and pearls most relevant for residents day-to-day practice. This podcast should serve as a distinct, pragmatic source in the context of other senior educators who may also teach about this topic.*

*Because we are residents (not content experts) aiming to educate our peers, a dialogue between two speakers can effectively model the collaborative tone intended for this project. It may also have entertainment value given that individually each of us are inexperienced with producing engaging audio recordings.*

- How do the learning objectives create a framework for organizing the educational content?

*The objectives are designed to inform the care of a patient on initial evaluation, so the framework will be the timeline of this assessment and actions with educational commentary to accompany each decision as needed.*

**Tasks to complete:**

- Utilize one or more narrative arcs to organize the educational content by following the course of a patient, a clinician, or a learner.

*We will use a mock-narrative of a resident responding to an urgent page about loss of bright red blood. The resident character will verbalize their thoughts and behavior with the other speaker generally behaving as a just-in-time educator for those decisions.*

- Find or create opportunities for spaced repetition of key concepts.

*At a minimum, we will highlight learning objects at the introduction, at the point where they occur in the lesson, and at the end.*

- Design an introduction and conclusion for engaging the audience, highlighting learning objectives, and thanking contributors.

*Theme song for intro and outro is chosen from royalty-free online sources.*

- Identify a senior advisor with expertise in the topic.

*We initially ask recognized expert medical educators with whom we have existing relationships.*

- Send script drafts to senior advisor for revision and review.
- Incorporate feedback into the script draft, repeat process for further review as needed.
- Continue iterative process until satisfactory script has been created.

**Tangible product:**

- Final script to be used for recording

**4) Recording Audio**

**Questions to ask:**

- What tone do you want to set?

*We want a conversational tone with an emphasis on the “back-and-forth" dynamic between the two hosts as well as some ad-hoc humor. We will portray ourselves as knowledgeable on the topic but not experts.*

- What is your budget for audio equipment and processing?

*Our budget is under $100, with the majority of the cost attributable to the microphone. Episodes will be recorded in our homes or conferences rooms in the hospital. Episodes will be recorded using the free recording platform Audacity.*

**Tasks to complete:**

- Practice out-loud and revise script to suit spoken language and refine delivery
- Identify areas poorly suited for the audio format (such as lists or dense content) and revise (consider utilizing a framework)

*We will not list the full American Trauma Life Support staging criteria for hemorrhagic shock. Instead, we will discuss key elements of those criteria as broader concepts used to determine patient stability. We will not list peripheral IV or central line flow rates. but emphasize the principle that large bore IVs are superior for resuscitation to a central line. We also will not give University of Washington specific color-coding for IVs, but encourage the listener to learn the system at their own institution.*

- Send audio recording to expert clinician for final approval
- Use audio post-production tools to remove unwanted sounds, level audio and increase overall podcast polish

*We will utilize the program Auphonic for audio leveling, in addition to the editing of audio within Audacity.*

**Tangible Product:**

- Finalized recording of episode ready for dissemination

**5) Releasing a podcast**

**Questions to ask:**

- What will be used to measure success?

*Our goal is to demonstrate the podcasts are liked and consistently utilized. As such, we track listenership data over time. Trainees will also be sent pre and post surveys related to enjoyment of the podcast and recommendations on how they could be improved.*

- What is the material use-case for listeners of this podcast?

*Listeners can access the episodes at any time via our blog, RSS feed, or Itunes. The access will not be restricted in any way which will reduce barriers to utilization but does not data tracking more challenging. The episodes are outside the structure of any planned educational activities at the University of Washington. They are standalone educational experiences that are not part of a larger coursework.*

**Tasks to complete:**

- Choose a podcast hosting site to suit needs and upload audio files. Consider whether the platform will allow it to be searchable on iTunes, hosted on institutional servers, or password protected.

*Blubrry will be utilized as the hosting platform, with an associated Wordpress blog, RSS feed and Itunes profile.*

- Distribute announcements about this new content: in person, e-mail, blog post, or social media.

*New episodes will be announced to residents via email, as well as automatic notifications via Itunes and the RSS feed for those already subscribed.*

**Tangible product:**

- Finished audio file is available for download or streaming in web browser or application.
