# Supplementary material for: How to Create and Evaluate a Resident-Led Audio Program: Six Clinical Podcasts for Medicine House Staff
Source: MedEdPORTAL. 2020 Dec 30;16:11062. doi: 10.15766/mep_2374-8265.11062 (PMC7780742; doi:10.15766/mep_2374-8265.11062)
Supplement: Supplementary file 1 — Needs Assessment Questionnaire.docxPre- and Postsurveys.docxDevelopers Guide.docxCompleted Developers Guide.docxGI Bleed.mp3SVT.mp3Toxidromes Part 1.mp3Transfusion Reactions.mp3Hypoxemic Respiratory Failure.mp3WCT.mp3 [file mep_2374-8265.11062-s001.zip › _Educational_Summary_Report_11062.pdf]

MedEdPORTAL® | The AAMC Journal of Teaching and Learning Resources

Original Publication

Open Access

How to Create and Evaluate a Resident-Led Audio Program: Six Clinical Podcasts for Medicine House Staff

Christopher Ghiathi, MD*, Kevin Seitz, MD, MSc, Patricia Kritek, MD, EdM

*Corresponding author: cghiathi@gmail.com

Abstract

Introduction: Podcasting in medical education has grown substantially. However, podcasts focused on internal medicine topics are relatively uncommon, and those created by or designed for medicine residents are rare. We investigated the feasibility and utilization of an open-access resident-created podcast targeted to the educational needs of internal medicine house staff. **Methods:** We distributed a needs assessment to 184 internal medicine residents at the University of Washington to assess podcast preferences and clinical scenarios perceived to be challenging. Based on the results, we developed a standardized method for podcast development and production. We created six episodes, utilizing a web-based podcasting platform. For outcome measures, we collected the number of unique downloads, and the perception of the podcast was evaluated by residents in comparison to other residency-sponsored educational activities with a survey. **Results:** Eighty-one residents (44%) completed the needs assessment, with participants expressing interest in resident-focused podcasts and a preference for relatively short episodes focused on high-yield clinical information. The episodes were downloaded 661 times. Residents gave the podcast an average rating of 4.32 out of 5 ($n = 22$), among the highest of educational modalities surveyed. Our podcasting development process also resulted in a generalized, reusable schema. **Discussion:** Our resident-generated podcasts were desired, feasible, and well utilized. They were also rated highly compared to more traditional educational modalities. Our podcast-creation schema serves as a road map for trainees to develop podcasts. Podcasting can be a resource for resident education and an opportunity for residents to grow as medical educators.

Keywords
Podcast, Peer Education, Curriculum Development, FOAM, Free Open-Access Meducation, Educational Technology, Internal Medicine

Educational Objectives

By the end of this activity, learners will be able to:

1. Evaluate characteristics for medical education podcasts desired by a target audience of internal medicine trainees.
2. Design, produce, record, and distribute a medical education podcast to a target audience.
3. Describe the evaluation, workup, and initial management of common inpatient scenarios.

Introduction

Digital and mobile resources for medical education have proliferated and diversified in form and content, with medical podcasting growing substantially over the last 10 years.[1,2] Podcasting has demonstrated effectiveness as a primary mode of medical education, comparable to conventional lectures or text-based resources.[3,4] Podcasts have also been studied as a flexible and effective supplement to other educational modalities.[5-7] Podcasts have been successful in part because they are perceived by listeners to be efficient, allowing them to multitask while increasing their medical knowledge, and are easily accessible on mobile platforms.[7] Over the last decade, the need for best practices to guide medical education podcasting is receiving increased attention from peer-reviewed publications and national organiations.[8,9] Surveys of podcast content and structure across subspecialties demonstrate that podcasts are currently most common in the fields of anesthesia and emergency medicine.[10,11]

As learners developing practical clinical knowledge and skills under significant time constraints, resident physicians have unique educational needs.[12,13] Podcasts address these needs by utilizing asynchronous learning to deliver targeted content

Citation:
Ghiathi C, Seitz K, Kritek P. How to create and evaluate a resident-led audio program: six clinical podcasts for medicine house staff. *MedEdPORTAL.* 2020;16:11062.
https://doi.org/10.15766/mep_2374-8265.11062

Copyright © 2020 Ghiathi et al. This is an open-access publication distributed under the terms of the Creative Commons Attribution license.

in a flexible and accessible format.[10,14,15] They are also popular among residents as an educational modality.[16] Almost 28,000 residents are currently in internal medicine training programs, and while there are popular and well-established podcasts within the specialty, podcasts focused on internal medicine topics remain relatively uncommon.[11,17] Podcasts created by or specifically targeting medicine residents are rare.[18,19]

In residency training programs, senior residents educate their peers and junior residents in direct patient care, on daily rounds, and in teaching conferences; podcasting provides a modality to enhance this existing role as a peer-educator.[20] However, guidelines to inform the design of effective medical education podcasts are nascent, and a paucity of literature exists on how to address the particular needs of internal medicine residents with clinical education podcasts, much less for resident-led educational programs.[9,21-23] We investigated the desirability and feasibility of a free, open-access, resident-created podcast targeted to the unique educational needs of internal medicine house staff. In doing so, we developed a generalizable process map to guide the production of podcasts by others.

Methods

Needs Assessment

We sought to design podcasts that would be available independent of clinical rotations or existing educational curricula. No prerequisite knowledge was expected from learners beyond completion of undergraduate medical education. We identified a need in our residency program for education focused on care of the hospitalized patient, and we sought to determine if a podcast could fill that need. To identify the interests and preferences of the target audience, we distributed a needs assessment survey to all internal medicine residents at the University of Washington using the web-based REDCap data-collection software (Appendix A).[24] This survey consisted of seven questions regarding participant demographics, desire for a resident-specific medical podcast, podcast preferences, and clinical scenarios perceived to be challenging.

Podcast Design

In keeping with the established literature on the perceived benefits of podcasts, we wanted to ensure our episodes would deliver medical knowledge, could be enjoyed while multitasking, and were easily accessible. Guided by the results of the needs assessment, we developed a framework for podcast production that could provide a standardized and reproducible process map for planning and recording each episode (Figure 1). This approach began with the selection of a clinical topic that was informed by the interests of the residents captured in the needs assessment survey (in Figure 1: Topic). To focus these topics, we identified a specific clinical scenario for each. After a review and compilation of relevant educational content from trusted resources (in Figure 1: Content), we wrote a podcast script draft (in Figure 1: Script). Our scripts focused on the initial assessment,

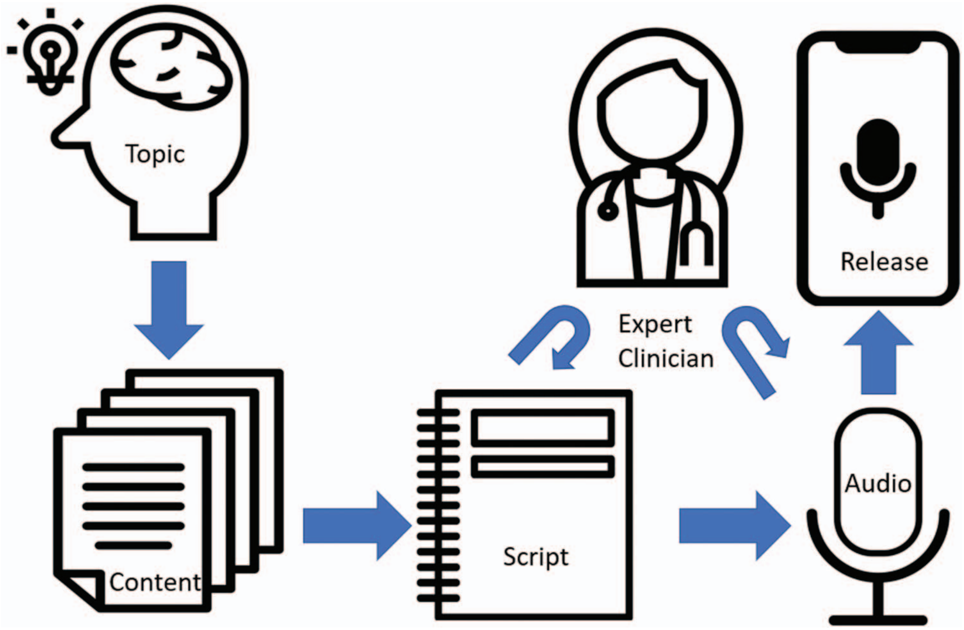

Figure 1. Visualization of the podcast-creation process.

Copyright © 2020 Ghiathi et al. This is an open-access publication distributed under the terms of the Creative Commons Attribution license.

triage, diagnostics, and therapeutics of the clinical scenario. We delineated learning objectives and emphasized key concepts with spaced repetition. Next, we digitally transmitted our script draft to a faculty attending physician chosen for expertise related to the topic (in Figure 1: Expert Clinician) for review and revision. This iterative feedback process was continued until a final script was complete and satisfactory.

Following completion of the script, we recorded an initial oral version of the episode (in Figure 1: Audio). This performance was recorded on a USB microphone using a free and open-source digital audio recording and editing application software (Audacity version 2.2.0). This recording was reviewed, and additional recordings were made until an acceptable recording was captured. Next, we sent the initial digital recording back to the previously identified expert clinician for any further guidance or revisions. The audio file was edited for length and refined with an audio file processor (Auphonic) to reduce background noise and correct volume and stereo level differences. We uploaded to a podcast hosting platform (Blubrry RawVoice) the finalized digital audio file of the episode (in Figure 1: Release). Once the episode had been uploaded, we published a hyperlink for it on an associated blog (WordPress) to facilitate distribution via web-based platforms such as an RSS feed and iTunes. In addition, we disseminated a hyperlink to each new episode by email to all internal medicine residents. The podcast hosting service tracked individual user access and provided quantitative summary data on number of listens and type of device (mobile phone applications, web browsers, etc.) utilized for listening.

Through producing and releasing each episode, we revised our production process as a podcast developer's guide with five sequential stages (Appendix C). These stages represent discrete and sequential steps in the podcast-creation process. For each stage (Topic, Content, Script, Audio, and Release), we provide prompts designed to help generate content and specific tasks for iterative improvement. Addressing the question prompts at each stage and completing the tasks as described will result in a well-defined, tangible end product, which is necessary for beginning the following stage. A final version of the developer's guide can be found in Appendix D, accompanied by our responses to each question illustrating the development of our first episode.

We released a total of six episodes (Appendices E-J) using the podcast-creation schema detailed in Figure 1 over the course of 18 months. Of those six, "Transfusion Reactions," "Toxidromes and Overdoses," "Hypoxemic Respiratory Failure," and "Wide Complex Tachycardia" were chosen as episodes based on the results of the needs assessment. "Acute Gastrointestinal Bleed" and "Supraventricular Tachycardia" were chosen based on our own preferences. Christopher Ghiathi and Kevin Seitz created all six episodes.

Evaluation

The effectiveness of this project was assessed in two ways. Specific to each episode, we created surveys in REDCap, a web application for survey distribution and data management, to assess comfort with the clinical content before and after listening (Appendix B). These pre- and postlisten surveys were sent by email and released concurrently with each of the first four episodes. Residents were asked to rate their perceptions of understanding, confidence in management, and comfort with teaching the given topic on a 5-point Likert scale (1 = *Strongly disagree,* 5 = *Strongly agree*). Verbal reminders to complete the pre- and postlisten surveys were included at the start of each podcast audio file.

Finally, we included the podcast on a yearly programmatic survey of educational modalities at the University of Washington internal medicine residency program to capture perceptions of educational utility compared to other resources. All educational modalities, including this podcast program, morning report, grand rounds, and other institution-sponsored conferences, were evaluated on a 5-point Likert scale (1 = *Poor,* 5 = *Excellent*).

Results

In total, 81 internal medicine residents (44%) at University of Washington completed the needs assessment. Thirty-three respondents (41%) to the needs assessment were interns. Only 38% of all residents listened to any medical podcast programs at the time of the survey, but nearly all respondents (99%) said they would utilize a podcast designed for internal medicine house staff (Table 1). The majority of participants (64%) identified 10-20 minutes as the ideal length for a podcast, and 98% of those surveyed identified high-yield clinical pearls as an important feature in a medical podcast.

As of November 6, 2019, the six episodes have been downloaded 661 times, with each episode ranging from 67 to 141 downloads. Of all downloads, 462 (70%) were via a mobile phone application (Figure 2). The number of unique listeners, however, cannot be quantified with the sources of data available. Pre- and postlisten evaluations were distributed with the first four podcasts. Twenty-three residents completed prelisten surveys, with only seven completing both the pre- and postlisten surveys.

Sixty-nine University of Washington internal medicine residents completed the 2019 curricular evaluation survey distributed by the residency program (Table 2). Of those respondents, 22 (32%)

Copyright © 2020 Ghiathi et al. This is an open-access publication distributed under the terms of the Creative Commons Attribution license.

MedEdPORTAL® The AAMC Journal of Teaching and Learning Resources

Table 1. Resident Interest in and Preferences Regarding Medical Podcasts

Survey Question and Response Choices	No. (%) Residency Year 1 (*n* = 33)	Residency Years 2-4 (*n* = 48)	Residency Years 1-4 (*n* = 81)
What do/would you appreciate most in a medical podcast?			
Clinical pearls.	32 (97)	47 (98)	
References to key literature.	18 (54)	23 (48)	
Expert opinion.	16 (48)	17 (35)	
Latest or emerging innovations.	8 (24)	19 (40)	
Other.	0 (0)	1 (2)	
I see no role for medical podcasts in my life.	0 (0)	1 (2)	
How many hours per week to you listen to podcasts?			
I don't routinely listen to podcasts.	7 (21)	23 (48)	
Less than 2 hours.	13 (39)	15 (31)	
2-4 hours.	12 (36)	6 (12)	
More than 4 hours.	1 (3)	4 (8)	
Do you listen to any medical podcasts?			
Yes	14 (42)	17 (35)	
No	19 (58)	31 (65)	
Would you utilize a podcast series designed for internal medicine residents focused on the essentials of managing topics like those mentioned above?			
Yes	33 (100)	47 (98)	
No	0 (0)	1 (2)	
What is the ideal length of a podcast in your life?			
I don't routinely listen to podcasts.			13 (16)
Less than 2 hours.			52 (64)
2-4 hours.			9 (11)
More than 4 hours.			7 (9)

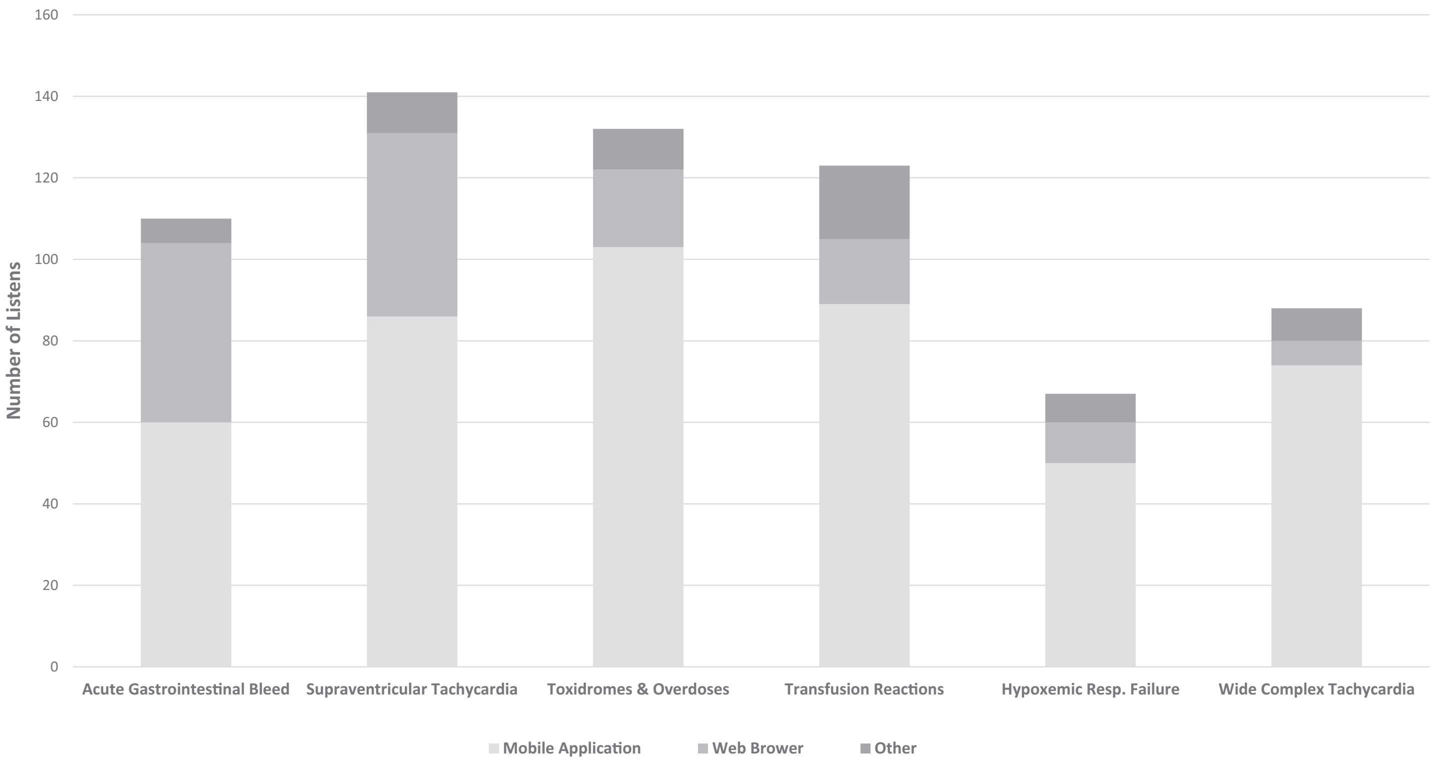

Figure 2. Number of listens for each podcast episode with the associated distribution of devices used. Episodes are listed in order of release.

Copyright © 2020 Ghiathi et al. This is an open-access publication distributed under the terms of the Creative Commons Attribution license.

Table 2. Residents' (n = 69) Likert-Scale Ratings of Educational Activities

Educational Activity	1 (Poor)	2 (Fair)	3 (Good)	4 (Very Good)	5 (Excellent)	N/A	*M*	*SD*
Thursday morning academic half-day conferences	3%	9%	22%	45%	28%	0%	3.74	1.0
Monday lunch core teaching conferences	2%	0%	19%	33%	36%	10%	4.15	0.9
Attending teaching rounds on ward rotations	2%	2%	19%	44%	35%	0%	4.09	0.8
Morning report conferences	2%	2%	9%	28%	61%	0%	4.45	0.8
Department of Medicine grand rounds	2%	12%	26%	36%	17%	7%	3.61	1.0
Other conferences (e.g., chief of medicine)	0%	2%	14%	33%	42%	9%	4.27	0.8
Podcasts ("Chris and Kevin's Bedside Chats")	0%	2%	3%	12%	16%	68%	4.32	0.8
Other online/electronic resources	0%	4%	10%	17%	19%	46%	4.00	1.0

evaluated the podcast with a mean score of 4.32 out of 5, as 19 (86%) rated the podcast as excellent or very good. These ratings were higher on average than ratings for University of Washington Department of Medicine grand rounds ($M = 3.61$, $SD = 1.0$, $n = 64$), teaching by attending physicians on rounds ($M = 4.09$, $SD = 0.8$, $n = 69$), or weekly 1-hour didactic series on core internal medicine content ($M = 4.15$, $SD = 0.9$, $n = 62$). The mean evaluation score for the podcast was lower than that for morning report ($M = 4.45$, $SD = 0.8$, $n = 69$).

Discussion

To address perceived gaps in resident education at our training program, we developed an open-source podcast series. Using a combination of surveys of resident perception and tracking of total downloads, we demonstrated the desirability, feasibility, and consistent utilization of a medical education podcast created by residents for their peers. With those results, we have presented a schema to guide the development of podcasts by other residents, attentive to their local needs and opportunities. This work adds to existing knowledge about medical education podcasting in two ways. First, we have characterized the desirability for this medical education format among internal medicine residents. A growing body of research has been published to describe the podcast listening habits and preferences of trainees, particularly within the disciplines of anesthesia and emergency medicine.[11-13] Few medical education podcasts provide content specifically developed for internal medicine trainees, and there has been minimal evaluation published to describe these programs in general internal medicine. In our needs assessment and monitored distribution, we found this content and format were highly desirable. Residents clearly identified their preferences regarding medical education podcasting and engaged with the content consistently over the course of 18 months. The podcast was also well liked by residents.

Second, we have described a generalizable approach to the development and production of a medical education podcast by resident trainees for their peers. The body of literature regarding best practices in podcast creation and evaluation is growing. There is minimal literature, however, on techniques and tools for residents to create podcasts for their peers. We developed and utilized a structured approach for producing podcasts that can reduce barriers for resident participation in this educational format. The use of our process map schema provides a step-by-step guide for residents to create their own podcasts, with a generalized framework adaptable to other settings and clinical topics. The questions at the initiation of each stage provide instruction for residents to determine the scope, style, and focus of their podcast. The iterative tasks outlined at each stage ensure content quality both in delivering educational value and in meeting the needs of the target audience. Each stage advances the work in a stepwise fashion through an additive and constructive process for a targeted and effective episode.

Reflecting on what we learned developing our podcast, we have identified several key aspects to make this type of intervention successful. In our opinion, performing a formalized needs assessment of the target audience is a critical first step. Trainees' preferences regarding medical podcasts may differ across specialties, institutions, and stage of training. Obtaining preferences from the target audience offers valuable information and yields focus and specific target parameters to guide development of the podcast. Those preferences were essential to developing our episode ideas, defining our objectives, and generating our scripts. It is also important to have a strategy for getting trainees to buy into using the podcast. We utilized a strategy of in-person advertisement at residency program conferences, as well as electronic invitations. These steps were important for reaching our target audience and maintaining listeners over time. We also recognize that podcasts, like all open-source education, do not undergo a traditional peer review process. Thus, it is imperative that episode content references the medical literature, and we encourage listeners to use multiple information sources when deciding on best practice.

The most significant limitation to our study is the evaluation of educational effectiveness. Overall, completion rates for pre- and postlisten surveys of each episode were consistently low,

Copyright © 2020 Ghiathi et al. This is an open-access publication distributed under the terms of the Creative Commons Attribution license.

even with reminders by email and prompts within the audio recordings. By design, these podcasts were released with the aim of minimizing barriers to use, being available at any time and accessible without password protection or requiring survey completion. As a result, we feel our approach led to higher overall utilization and uptake in the residency program. The number of listens and survey results about these podcasts in aggregate were used as proxy measures of educational effectiveness. However, our study could have had increased survey completion with a more structured dissemination approach, providing higher yield of data collection. Furthermore, design and production of podcasts would be enhanced by receiving iterative feedback from end users to guide continuous improvement efforts, including the opportunity to capture whether the listeners are, in fact, junior residents, as we intended. A standardized process for feedback delivery such as a rubric from faculty to the podcast creators would also enhance the educational experience. Another limitation to the interpretation of our results is that we were able to collect only the total number of downloads and were unable to track the number of unique listeners or whether the podcast was downloaded by the same person multiple times.

From this experience, we believe that the next step in evaluating medical education podcasting is to transition from reactions-focused to learning-focused outcomes data. Our study supports the findings demonstrated in prior research that podcasts are popular among residents, including when they are created by their peers. However, we were not able to assess if there was a change in the knowledge, skills, or attitudes of those who listened to our podcasts. As discussed, we experienced several challenges in using surveys to obtain this information, and we see several ways in which future medical education research could address them. Integrating a podcast program into the established curriculum of residency programs may allow for protected time to complete the surveys. Completion of pre- and postlisten surveys could be required in the context of the educational expectations of a course or rotation. Alternatively, because surveys have limitations in assessing learning outcomes, future work could evaluate the use of resident-created podcasts as a primer to other educational activities, including flipped classroom models and simulation, or programmatic assessments, such as in-service exams.

We feel that educational content created by trainees not only provides unique educational content for listeners but also enhances skills for podcast authors in a format with significant potential for growth. Peer-generated medical education podcasts provide an opportunity for residents to learn from and teach each other. The residents creating the podcasts begin the process as learners and, with guidance from faculty, are able to increase their own topic competency. In addition to their needs as learners, medical residents have roles as educators, and podcasting provides an opportunity to develop skills as teachers while also creating tangible educational products. We have developed a podcast-creation schema that provides a road map for other residents to create content. Going forward, this type of education can be expanded into a platform for trainees to generate their own content and share it with their peers. This strategy would require the use of our podcast-creation schema and mentoring of new contributors. Once they are familiar with the process, multiple residents could develop topics and create episodes independently on a common hosting platform. Ideally, this process would result in a cumulative repository of curricula and provide more opportunities for asynchronous learning that could be integrated in resident education.

Appendices

A. Needs Assessment Questionnaire.docx
B. Pre- and Postsurveys.docx
C. Developers Guide.docx
D. Completed Developers Guide.docx
E. GI Bleed.mp3
F. SVT.mp3
G. Toxidromes Part 1.mp3
H. Transfusion Reactions.mp3
I. Hypoxemic Respiratory Failure.mp3
J. WCT.mp3

All appendices are peer reviewed as integral parts of the Original Publication.

Christopher Ghiathi, MD: Chief Resident, Department of Medicine, VA Puget Sound Healthcare System and University of Washington School of Medicine; ORCID: https://orcid.org/0000-0001-7688-439X

Kevin Seitz, MD, MSc: Chief Resident, Department of Medicine, Harborview Medical Center and University of Washington School of Medicine

Patricia Kritek, MD, EdM: Professor, Department of Medicine, University of Washington School of Medicine

Disclosures
None to report.

Copyright © 2020 Ghiathi et al. This is an open-access publication distributed under the terms of the Creative Commons Attribution license.

Funding/Support
None to report.

Prior Presentations
Ghiathi C. Housestaff podcasting from bedside to headphones: a needs assessment and feasibility pilot for an education audio program. Presented at: WA-ACP Scientific Scholarship Day, May 18, 2018; Seattle, WA.

Ethical Approval
The University of Washington Human Subjects Division approved this study.

References

1. Cadogan M, Thoma B, Chan TM, Lin M. Free Open Access Meducation (FOAM): the rise of emergency medicine and critical care blogs and podcasts (2002–2013). *Emerg Med J*. 2014; 31(e1):e76-e77. https://doi.org/10.1136/emermed-2013-203502
2. Kyaw BM, Saxena N, Posadzki P, et al. Virtual reality for health professions education: systematic review and meta-analysis by the Digital Health Education Collaboration. *J Med Internet Res*. 2019;21(1):e12959. https://doi.org/10.2196/12959
3. Schreiber BE, Fukuta J, Gordon F. Live lecture versus video podcast in undergraduate medical education: a randomised controlled trial. *BMC Med Educ*. 2010;10:68. https://doi.org/10.1186/1472-6920-10-68
4. Narula N, Ahmed L, Rudkowski J. An evaluation of the "5 Minute Medicine" video podcast series compared to conventional medical resources for the internal medicine clerkship. *Med Teach*. 2012;34(11):e751-e755. https://doi.org/10.3109/0142159X.2012.689446
5. Chin A, Helman A, Chan TM. Podcast use in undergraduate medical education. *Cureus*. 2017;9(12):e1930. https://doi.org/10.7759/cureus.1930
6. Prakash SS, Muthuraman N, Anand R. Short-duration podcasts as a supplementary learning tool: perceptions of medical students and impact on assessment performance. *BMC Med Educ*. 2017;17:167. https://doi.org/10.1186/s12909-017-1001-5
7. Malecki SL, Quinn KL, Zilbert N, et al. Understanding the use and perceived impact of a medical podcast: qualitative study. *JMIR Med Educ*. 2019;5(2):e12901. https://doi.org/10.2196/12901
8. Sandars J. Twelve tips for using podcasts in medical education. *Med Teach*. 2009;31(5):387-389. https://doi.org/10.1080/01421590802227958
9. Lin M, Thoma B, Trueger NS, Ankel F, Sherbino J, Chan T. Quality indicators for blogs and podcasts used in medical education: modified Delphi consensus recommendations by an international cohort of health professions educators. *Postgrad Med J*. 2015; 91(1080):546-550. https://doi.org/10.1136/postgradmedj-2014-133230
10. Cho D, Cosimini M, Espinoza J. Podcasting in medical education: a review of the literature. *Korean J Med Educ*. 2017;29(4):229-239. https://doi.org/10.3946/kjme.2017.69
11. Little A, Hampton Z, Gronowski T, Meyer C, Kalnow A. Podcasting in medicine: a review of the current content by specialty. *Cureus*. 2020;12(1):e6726. https://doi.org/10.7759/cureus.6726
12. DaRosa DA, Skeff K, Friedland JA, et al. Barriers to effective teaching. *Acad Med*. 2011;86(4):453-459. https://doi.org/10.1097/ACM.0b013e31820defbe
13. Sklar DP. Just because I am teaching doesn't mean they are learning: improving our teaching for a new generation of learners. *Acad Med*. 2017;92(8):1061-1063. https://doi.org/10.1097/ACM.0000000000001808
14. Matava CT, Rosen D, Siu E, Bould DM. eLearning among Canadian anesthesia residents: a survey of podcast use and content needs. *BMC Med Educ*. 2013;13:59. https://doi.org/10.1186/1472-6920-13-59
15. Mallin M, Schlein S, Doctor S, Stroud S, Dawson M, Fix M. A survey of the current utilization of asynchronous education among emergency medicine residents in the United States. *Acad Med*. 2014;89(4):598-601. https://doi.org/10.1097/ACM.0000000000000170
16. Riddell J, Swaminathan A, Lee M, Mohamed A, Rogers R, Rezaie SR. A survey of emergency medicine residents' use of educational podcasts. *West J Emerg Med*. 2017;18(2):229-234. https://doi.org/10.5811/westjem.2016.12.32850
17. Number of programs and residents. American Board of Internal Medicine. 2020. https://www.abim.org/about/statistics-data/resident-fellow-workforce-data/number-of-programs-residents.aspx
18. Duckworth S, Leung TI, O'Glasser A, Clark A. Listen and learn: a guide to podcasts for internists. *SGIM Forum*. 2019;42(7):1-3. https://www.sgim.org/File%20Library/SGIM/Resource%20Library/Forum/2019/SGIM-Jul-8.pdf
19. Wolpaw J, Toy S. Creation and evaluation of an anesthesiology and critical care podcast. *J Educ Perioper Med*. 2018;20(1):E620. https://www.ncbi.nlm.nih.gov/pmc/articles/PMC5991777/
20. Qian ET, Leverenz DL, McPherson JA, Kroop SF. An internal medicine residency podcast: impact on the educational experience and care practices of medical residents. *J Gen Intern Med*. Published online June 10, 2020. https://doi.org/10.1007/s11606-020-05939-3
21. Ahn J, Inboriboon PC, Bond MC. Podcasts: accessing, choosing, creating, and disseminating content. *J Grad Med Educ*. 2016;8(3):435-436. https://doi.org/10.4300/JGME-D-16-00205.1
22. Lin M, Joshi N, Grock A, et al. Approved Instructional Resources Series: a national initiative to identify quality emergency medicine blog and podcast content for resident education. *J Grad Med Educ*. 2016;8(2):219-225. https://doi.org/10.4300/JGME-D-15-00388.1

Copyright © 2020 Ghiathi et al. This is an open-access publication distributed under the terms of the Creative Commons Attribution license.

23. Singh D, Alam F, Matava C. A critical analysis of anesthesiology podcasts: identifying determinants of success. *JMIR Med Educ*. 2016;2(2):e14. https://doi.org/10.2196/mededu.5950

24. Harris PA, Taylor R, Thielke R, Payne J, Gonzalez N, Conde JG. Research electronic data capture (REDCap)—a metadata-driven methodology and workflow process for providing translational research informatics support. *J Biomed Inform*. 2009;42(2): 377-381. https://doi.org/10.1016/j.jbi.2008.08.010

Received: March 5, 2020
Accepted: September 15, 2020
Published: December 30, 2020

Copyright © 2020 Ghiathi et al. This is an open-access publication distributed under the terms of the Creative Commons Attribution license.
